# Medication transcription errors in hospitalized patient settings: a consensual study in the Palestinian nursing practice

**DOI:** 10.1186/s12913-019-4485-3

**Published:** 2019-09-06

**Authors:** Ramzi Shawahna, Abbas Abbas, Ameed Ghanem

**Affiliations:** 1 Department of Physiology, Pharmacology and Toxicology, Faculty of Medicine and Health Sciences, An-Najah National University, New Campus, Building: 19, Office: 1340, P.O. Box 7, Nablus, Palestine; 2 An-Najah BioSciences Unit, Centre for Poisons Control, Chemical and Biological Analyses, An-Najah National University, Nablus, Palestine; 3 Department of Medicine, Faculty of Medicine and health Sciences, An-Najah National University, Nablus, Palestine

**Keywords:** Delphi technique, Nurse, Medication errors, Medication transcription errors, Palestine

## Abstract

**Background:**

Medication transcription errors (MTEs) are frequent in hospitalized patient settings. Definitions and scenarios that represent potential MTEs in the Palestinian nursing practice were not previously approached using formal consensus techniques. This investigation was conducted to develop a consensual definition of MTEs and scenarios that represent different MTE situations by a panel of nurses and other healthcare professionals.

**Methods:**

In this observational study, consensus was sought using the Delphi technique. Panelists (*n = 64*) were invited and recruited from different hospitals in Palestine and a two-iterative rounds Delphi technique was used to achieve consensus on a proposed definition of MTEs and 76 different scenarios representing potential MTEs.

**Results:**

Consensus was achieve to accept the definition and to consider 69 of the 76 proposed scenarios (77.6%) as MTEs, exclude 3 scenarios (3.9%), and 4 scenarios (5.3%) remained equivocal. Equivocal scenarios might be considered as MTEs or not depending on the clinical situation.

**Conclusions:**

Consensus was achieved on a definition of MTEs and scenarios representing MTEs by a panel of nurses and other healthcare professionals. This study showed that it was possible to develop and achieve consensus on a definition and scenarios representing MTE situations using formal consensus techniques. Such consensual definitions could be useful in future epidemiological studies investigating MTEs. Using consensual definitions might reduce methodological variations, promote congruence in error counting and reporting, and permit comparing error rates in different hospital settings.

**Electronic supplementary material:**

The online version of this article (10.1186/s12913-019-4485-3) contains supplementary material, which is available to authorized users.

## Background

Despite the advancing leaps of modern healthcare delivery, hospitals remain a source of harm to patients rather than places of hoped healing [1]. The Institute of Medicine published its famous report *Err is Human: Building a Safer Health System* in the year 2000 and since then medication errors have captured the attention of health authorities and research around the world [2, 3]. Subsequently, reduction of medication errors has emerged as a top priority in different healthcare systems around the globe. Hospitalized patients are at a particularly increased risk of being harmed by medication errors compared to other patients as a result of their illnesses, fragility, and the number of medications they are taking [4].

Although the medication process has been computerized in some healthcare systems, the vast majority of the medication documentation around the globe is predominately handwritten and paper-based [5, 6]. In these healthcare systems, physicians prescribe medications either onto a specifically designated sheet in the inpatient’s profile or on separate paper sheets. Nurses then transcribe the medications prescribed by physicians on specific sheets (transcripts) to procure these medications either from a ward pharmacy or, in many cases, the central pharmacy of the hospital [5–7]. Upon arrival of these transcripts to the pharmacy, pharmacists dispense the corresponding volumes and doses of the prescribed medications. Medications are then distributed and administered to the patients concerned. Patients to whom medications were administered might be monitored for the therapeutic action of the medication, allergy, side effects, and/or medication’s concentration in their blood. Therefore, a medication error might take place during any phase of this multi-phase process. Medication errors occur during the prescription, transcription, dispensing, administration, and/or monitoring phase [5, 8–10]. Previous studies focused on the medication errors that occur during the prescription and/or administration stage [1, 7, 9–11]. Little attention was paid towards the errors occurring at the transcription stage.

Medication transcription errors (MTEs) are of particular importance because the different phases of prescription, transcription, dispensing, and administration occur in chain and, therefore, it is highly likely that if a medication was transcribed incorrectly, this error would go without interception and would most probably reach the patient and cause harm. Previous studies conducted in Switzerland, Pakistan, and Iran and showed that errors occurred at the medication transcription phase [5, 7, 11]. In a previous study conducted in Pakistan, MTEs occurred in 16.9 and 13.8% of the 6583 and 5329 medications transcribed onto inpatient profiles and discharge charts, respectively [5]. Pichon et al. conducted a study in Switzerland in which the nurses transcribed chemotherapy and non-chemotherapy related prescribed medications onto different sheets twice [7]. In the first transcription stage, 11.8 and 20.7% of the transcribed chemotherapy and non-chemotherapy medications, respectively, were incorrect. Fahimi et al. reviewed MTEs in a teaching hospital in Iran and MTEs occurred in about 30% of the 558 opportunities for errors [11]. It is noteworthy mentioning that in large hospitals where larger volumes of medications are prescribed which subsequently need to be transcribed before being dispensed and administered to patients, even lower error rates are of paramount importance given the increasing opportunities for errors and potential harms to the patients [12].

Medication errors are defined differently and many definition of medication errors were previously reported [13–15]. Obviously, methodological variation in defining what constitutes a medication error might have a significant impact on the error rates researchers disseminate in their studies reporting on medication errors [8, 9, 16, 17]. Researchers might use different definitions or scenarios representing medication error situations which ultimately would lead to variability in error rates reported in their studies. Therefore, defining medication errors is a step of paramount importance in analyzing the incidence and prevalence of medication errors in a particular setting. Formal consensus techniques have been used to reduce discrepancies in what constitutes a medication error and to achieve consensus on definitions and scenarios representing error situations. Formal consensus methods were used to define medication prescription, administration and dispensing errors [8, 9, 16, 17]. However, these techniques were not used to develop and achieve consensus on a definition of MTEs and scenarios that represent different MTE situations.

The aim of this study was to develop a definition of MTE and achieve consensus on this definition and the different scenarios that represent MTE situations by a panel of nurses and other healthcare professionals using the Delphi technique.

## Methods

### Defining MTEs and formulating scenarios representing error situations

Before attempting to define MTEs, the literature was searched for different definitions of MTEs. Previous studies defined MTEs as errors that occur at the transcription stage that involve deviation while transcribing medication orders from the previous prescribing step [5]. Another study defined MTEs as incomplete and/or wrong transcription of a medication order [7]. Garcia-Ramos and Baldominos Utrilla described MTEs as when the medication prescription did not match with what was transcribed on the nurse’s administration form [18]. In Fahimi et al., MTEs were defined as deviations in transcription of medication orders from the previous step, this could occur on an order sheet, notes, and/or documentations in the pharmacy database [11]. Finally, Lisby et al. defined MTEs as discrepancies in the names of the drugs, their formulations, routes of administration, doses, dosing regimens, omission of drugs, or addition of drugs which were not ordered or prescribed [19].

We approached and interviewed 10 key contact nurses with extensive experience in medication transcription. The key contacts were asked open-ended questions to define MTEs and provide potential MTE situations. Definitions and potential MTE situations were noted. The medication process in the Palestinian healthcare system as well as in the majority of healthcare systems in the world is still handwritten and paper-based. Therefore, we aimed to propose a definition of handwritten MTEs. Based on the previous definitions of MTEs found in the literature and the definitions proposed by the key contacts interviewed in this study, a definition of MTEs was rephrased and proposed. In this definition a handwritten MTE was defined as “*any discrepancy between the physician medication order and the medication order transcribed onto any document related to the patient concerned as the medical record, medication chart, medication request sheet, discharge medication chart and/or any other similar document*”.

We then conducted an extensive literature review to collect all potential MTE situations [5–7, 11, 14, 15, 19]. It is important to note also that we formulated some potential MTE situations based on other forms of medication errors like prescribing, administration, and dispensing errors [1, 4, 8–10, 12, 16, 17]. A total of 76 different potential MTE situation were collected. Potential MTE situations were transformed into scenarios and included into a questionnaire. The questionnaire was given to 5 healthcare professionals in a pilot to assess its readability and comprehensibility. The 5 healthcare professionals were not included in the subsequent Delphi rounds. Some scenarios were revised to enhance understanding as suggested by the healthcare professionals who took part in the pilot.

### The Delphi technique

In this study, we used the Delphi technique to develop and achieve consensus on a definition of MTEs and scenarios that represent different MTE situations. The Delphi technique was extensively used in developing and achieving consensus on inconclusive issues in healthcare [8, 9, 20–27]. Moreover, this technique was used in developing and achieving consensus on other forms of medication errors [8, 9, 16, 17]. The Delphi technique involves iterative rounds in which panelists express the degree to which they agree or disagree with items presented to them during the Delphi rounds. In each iterative round, panelists are given statistical summaries of the precedent round to help achieve consensus in the least number of rounds.

### Panelists

In this study, the panelists were purposively invited and recruited as in previous studies [8, 9, 16, 17]. As a prerequisite of the Delphi technique, the panelist should possess previous knowledge of the subject being investigated, therefore, in this study the panelists were chosen and invited on the merits of their academic qualifications and practical experience in the field of medication transcription. Because transcription of medication is within the purview of nurses, the majority of the invited and recruited panelists in this study was purposively nurses. The panelists were recruited from major governmental, private, and teaching hospitals in Palestine. Sixty four panelists were invited and recruited into the panel using personal contacts. The panel size used in this study was in the range of panels used in previous studies conducted to define other forms of medication errors [8, 9, 16, 17]. The inclusion criteria were as follows: 1) basic or advanced degree in one of the disciplines of healthcare (nursing, medicine, or pharmacy), 2) had a license to practice in Palestine, 3) been in practice for 5 or more years in a hospital setting, and 4) had been previously trained in minimization of medication errors and/or risk management. In this study, we intended to include panelists from both genders, representative of different age groups, living and practicing in different geographical locations, practicing in different settings, offering different services, having different clinical specialties, and having different hierarchal positions. This study was conducted without any incentives.

#### First iterative Delphi round

In the first iterative Delphi round, the questionnaires were delivered by hand to the 64 panelists. The questionnaires contained 4 parts. In the 1st part, the panelists were asked to give their demographic and practice details. In the 2nd part, the panelists were asked if: 1) their institutions surveilled MTEs, 2) their institutions encouraged reporting MTEs, 3) their current institutions regularly instructed/trained staff to minimize MTEs, and 4) they believed the instructions/training provided by their institutions were sufficient to reduce MTEs. The 3rd part contained the proposed definition of MTEs and panelists were asked to express the level of their agreement or disagreement with the proposed definition on a Likert scale of 9 points (1 indicated total disagreement and 9 indicated total agreement). The 4th part contained the 76 scenarios representing potential MTE situations and panelists were asked to express the level of their agreement or disagreement with each scenario on a Likert scale of 9 points (1 indicated total disagreement and 9 indicated total agreement). We encouraged the panelists to include written comments if they wished to justify and/or qualify their scores. The questionnaire used in the Delphi rounds is shown in Additional file 1 (additional files).

#### Analysis of the scores

Scores collected in the First Delphi round were entered in an EXCEL sheet (Microsoft Excel 2007) and their descriptive statistics were calculated. For all scores, the first quartile (Q1), median (Q2), third quartile (Q3), and interquartile (IQR) were computed. Scores were analyzed using the definitions specified in the previous studies [8, 9, 16, 17]. Briefly, consensus was said to have been achieved and the definition or scenario was rejected when the median score was in the range of 1–3 and the IQR was within the range of 0–2. Consensus was said to have been achieved and the definition or scenario was accepted when the median score was in the range of 7–9 and the IQR was in the range of 0–2. However, in case the median score was in the range of 4–6 or the IQR was more than 2, the definition or scenario was considered equivocal. Equivocal scenarios were included in the subsequent Delphi round.

#### Second Delphi round

In the second Delphi round, scenarios that were considered equivocal were included and the panelists were asked if they wished to reconsider their scores in view of the scores and comments of the other panelists. The panelists were reminded with their own scores given in the previous round, the median and IQR of the scores of other panelists and a summary of their comments. Scores obtained in the second Delphi round were analyzed using the same definitions of consensus used in the first Delphi round. We decided not to conduct a third Delphi round because it was highly likely that no consensus would be achieved as indicated from the scores and comments of the panelists.

### Ethical considerations

The ethical approval to conduct this study was obtained from the Institutional Review Board (IRB) Committee of An-Najah National University (Protocol approval # 121-Jan-2016). In this study, the panelists had to respond to items in a questionnaire, therefore, the IRB decided that a written consent was not necessary and panelists would need to provide verbal consent. All panelists provided verbal consents before participation. The Delphi technique is semi-anonymous technique in which the identity of the panelists is known to the researcher while each panelist remained anonymous to the remainder of the participants. Views and votes of panelists weighed equally during analysis.

## Results

### Demographic and practice details of the panelists

In this study, responses were obtained from the 64 panelists in the first Delphi round (response rate = 100%) and from 52 panelists in the second Delphi round (response rate = 81.3%). The detailed demographic and practice characteristics of all panelists are shown in Table 1.

**Table 1:** Demographic and practice details of the panelists (*n = 64*) who took part in the study

	Panelists	
	Number	Percentage of total (%)
Gender		
Male	35	54.7
Female	29	45.3
Age (years)		
24–35	39	60.9
36–49	17	26.6
50 and above	8	12.5
Qualifications		
Diploma	23	35.9
BSc	33	51.6
MD/PhD	8	12.5
Job title		
Head nurse	3	4.7
Staff nurse	51	79.7
Physician	8	12.5
Pharmacist/risk manager	2	3.1
Employer		
Governmental hospital	46	71.9
Private hospital	14	21.9
Teaching hospital	4	6.3
Experience (years)		
5–9	29	45.3
10–14	18	28.1
15 and above	17	26.6

*BSc* Bachelor of Science, *MD* Doctor of Medicine, *PhD* Doctor of Philosophy

Approximately 80% (51) of the panelists were staff nurses and 13% (8) were physicians. Around 72% (46) of the participants worked in governmental hospitals. Nearly 61% (39) belonged to the age group 24–35 years old and about 52% (33) had a BSc degree. About 55% (35) of the panelists were male and about 45% (29) had a number of years in practice in the range of 5–9 years.

### MTEs surveillance systems in their institutions

When asked if there was any medication surveillance system in their current institutions, approximately 60% (38) stated that such systems did not exist. Consistently, about 52% (33) stated that their institutions did not encourage reporting MTEs and about the same percentage indicated that they did not receive clear instructions and/or training on how to minimize MTEs in their institutions. Approximately 61% (39) of the panelists did not believe that instructions and/or training in place were enough to minimize MTEs. Table 2 shows the detailed distribution of their responses.

**Table 2:** Detailed distribution of responses of the panelists (*n = 64*) on items related to MTEs surveillance systems in their institutions

Item	Response distribution	
	n	%
Is there any MTE surveillance system in your current institution?		
Yes	26	40.6
No	38	59.4
Does your current institution encourage MTE reporting?		
Yes	31	48.4
No	33	51.6
Do you receive clear instructions and/or training from the management of your institution to minimize MTEs?		
Yes	31	48.4
No	33	51.6
Do you think that the instructions and/or training in place are enough to reduce MTEs?		
Yes	25	39.1
No	39	60.9

*MTEs* Medication transcription errors

### Definition of MTEs

When asked to indicate the degree to which they disagree or agree with the proposed definition of MTEs on a scale of 1–9, consensus was achieved to accept the proposed definition that a handwritten MTE can be defined as “any discrepancy between the physician medication order and the medication order transcribed onto any document related to the patient concerned as the medical record, medication chart, medication request sheet, discharge medication chart or any other similar document”. The median score was 9 and the IQR was 1.

### Scenarios that represent different MTE situations

Analysis of the scores obtained in the first Delphi round showed that consensus was achieved to consider 59 of the 76 potential scenarios as MTE situations. Analysis of the scores obtained in the second Delphi round showed that consensus was achieved to consider further 10 scenarios as MTE situations. The 69 of the 76 (77.6%) scenarios that were considered as MTE situations are shown in Table 3.

**Table 3:** Scenarios considered as MTE situations

#	Scenario	Round 1		Round 2	
		Median	IQR	Median	IQR
	*The nurse transcribed a different item than the one prescribed or failed to transcribe a prescribed item*				
1	Transcribing a completely different medication; e.g. ibuprofen instead of metronidazole	9.0	0.00	na	na
2	Transcribing a look-like medication; e.g. motilium instead of movalis	9.0	0.00	na	na
3	Transcribing a sound-like medication; e.g. sintrom instead of centrum	9.0	1.00	na	na
4	Failure to transcribe the dose of a medication that was prescribed to the patient	9.0	1.00	na	na
	*The nurse transcribed a different dose, frequency, dosage form or count of the prescribed item*				
5	Transcribing micrograms into milligrams or vice versa	9.0	2.00	na	na
6	Transcribing 0.x mL into x mL or vice versa	9.0	0.25	na	na
7	Transcribing x.0 mg or x.0 mL into ×0 mg or ×0 mL or vice versa	9.0	2.00	na	na
8	Transcribing a dose of medication that is lower than the dose prescribed	9.0	2.00	na	na
9	Transcribing a dose of medication that is higher than the dose prescribed	9.0	1.00	na	na
10	Transcribing a smaller number of dose units than prescribed	8.5	2.00	na	na
11	Transcribing a larger number of dose units than prescribed	9.0	1.00	na	na
12	Transcribing a dosage form that is different from the one prescribed; e.g. capsules instead of suppositories or vice versa	8.0	2.00	na	na
13	Transcribing a release form that is different from the one prescribed; e.g. a modified release formulation instead of conventional or vice versa	8.0	2.00	na	na
14	Failure to transcribe the frequency at which the drug was prescribed to be given	9.0	1.00	na	na
15	Transcribing a frequency of medication administration that is lower than the one prescribed; e.g. two times a day instead of three times a day	8.5	1.00	na	na
16	Transcribing a frequency of medication administration that is higher than the one prescribed; e.g. three times a day instead of two times a day	9.0	1.00	na	na
	*The nurse failed to transcribe or transcribed incorrect essential information related to the prescribed item*				
17	Failure to transcribe prescribed instructions to administer a medication in relation to meals	7.0	4.00	7.0	2.0
18	Transcribing prescribed instructions to administer a medication before meal for a medication that was prescribed to be administered after meal or vice versa	8.0	2.00	na	na
19	Failure to transcribe prescribed notes on how to administer a medication	8.0	2.00	na	na
20	Failure to transcribe the prescribed the maximum daily dose of a medication that was prescribed to be administered when required	8.0	2.00	na	na
21	Failure to transcribe prescribed notes pertaining to special warnings or precautions concerning the administration of a medication	8.0	2.00	na	na
22	Failure to transcribe prescribed special instructions pertaining to avoiding certain foods along with the prescribed medication	8.0	2.00	na	na
23	Failure to transcribe prescribed special instructions pertaining to avoiding certain drugs along with the prescribed medication	8.5	2.00	na	na
24	Failure to re-transcribe a medication order in full when a change has been made to it	9.0	2.00	na	na
25	Failure to transcribe prescribed instructions pertaining to when to start administering a medication	8.0	2.00	na	na
26	Failure to transcribe prescribed instructions pertaining to when to stop administering a medication	8.0	2.00	na	na
27	Failure to transcribe a prescribed medication	9.0	0.25	na	na
28	Transcribing a medication that was not prescribed	9.0	0.25	na	na
29	Transcribing a medication based on an expired medication order	9.0	1.00	na	na
30	Transcribing a medication that was put on hold	8.0	2.00	na	na
31	Transcribing a medication that was cancelled	9.0	1.00	na	na
32	Transcribing a wrong date	8.0	2.00	na	na
33	Transcribing a wrong ward	7.0	2.00	na	na
34	Transcribing a wrong patient name	9.0	0.00	na	na
35	Failure to transcribe prescribed special instructions pertaining to storage conditions of a medication	7.5	2.00	na	na
36	Failure to transcribe prescribed instructions to use special measuring tools to measure the dose of medication to be administered	8.0	2.00	na	na
37	Failure to transcribe prescribed special instructions on how to reconstitute (prepare) a medication	8.0	2.00	na	na
38	Failure to transcribe prescribed special instructions to disinfect a vial before administration of a medication	8.0	2.25	8.0	1.3
39	Transcribing a route of drug administration that is different to that prescribed	9.0	1.00	na	na
40	Transcribing a site of drug administration that was different to that prescribed	8.0	3.00	8.0	2.0
41	Transcribing a medication order twice	7.0	3.00	7.0	2.0
42	Transcribing a misspelled medication’s name (major), the medication can be confused with another	9.0	1.00	na	na
43	Transcribing a medication order illegibly	9.0	2.00	na	na
44	Transcribing a medication order using abbreviations or non-standard nomenclature	8.0	2.25	8.0	2.0
45	Failure to transcribe a prescribed duration for an intravenous infusion	8.0	2.00	na	na
46	In case the prescriber made a medication order for a patient on mg/kg basis and prescribed instructions to calculate the dose, failure to transcribe the finally calculated dose of medication on the patient’s documents	7.0	5.00	7.0	2.0
47	In case the prescriber made a medication order for a patient with renal impairment and prescribed instructions to calculate the dose, failure to transcribe the finally calculated dose of medication on the patient’s documents	9.0	1.00	na	na
48	Continuing to transcribe a medication for longer duration than instructed by the prescriber	8.0	2.00	na	na
49	Failure to transcribe prescribed notes on the presence of other clinical conditions that might affect decisions to administer future doses of the medication prescribed to the patient	8.5	2.00	na	na
50	Failure to transcribe prescribed information on the presence of allergies that might affect decisions to administer future doses of the medication prescribed to the patient	9.0	1.00	na	na
51	Failure to transcribe prescribed information on contraindications that might affect decisions to administer future doses of the medication prescribed to the patient	8.0	2.00	na	na
52	Failure to transcribe prescribed information on possibility of drug-drug interaction that might affect decisions to administer doses of the medication prescribed to the patient	8.0	2.00	na	na
53	Failure to transcribe prescribed instructions to avoid administering certain medications along with the prescribed medication to the patient	8.0	2.00	na	na
54	Failure to transcribe prescribed notes on possibility of drug-food interactions that might affect decisions to administer doses of the medication of the medication prescribed to the patient	8.0	2.00	na	na
55	Failure to transcribe prescribed instructions to avoid giving the patient certain foods along with the prescribed medication	8.0	2.00	na	na
56	Failure to transcribe special prescribed instructions on when to administer a medication; e.g. instructions to administer a dose of medication when the patient’s blood pressure drops below a certain limit	9.0	1.00	na	na
57	Failure to transcribe special prescribed instructions on when not to administer a medication; e.g. instructions not to administer a dose of medication when the patient’s heart rate drops below a certain limit	9.0	0.25	na	na
58	Failure to transcribe special prescribed instructions on monitoring the patient for certain signs before or after administering a medication; e.g. certain adverse effects	9.0	2.00	na	na
59	Failure to transcribe special prescribed instructions on using appropriate diluents in the preparation of a dose of a medication	8.0	2.00	na	na
60	Failure to transcribe special prescribed instructions on using appropriate solvents in the preparation of a dose of a medication	8.0	2.00	na	na
61	Failure to transcribe special prescribed instructions to avoid mixing physically incompatible ingredients for the preparation of a dose of a medication	8.0	2.00	na	na
62	Failure to transcribe special prescribed instructions to avoid mixing chemically incompatible ingredients for the preparation of a dose of a medication	8.0	2.00	na	na
63	Failure to transcribe special prescribed instructions to avoid subjecting light-sensitive ingredients to light during the preparation of a dose of a medication	8.0	2.00	na	na
64	Failure to transcribe special prescribed instructions to avoid subjecting heat-sensitive ingredients to heat during the preparation of a dose of a medication	8.0	2.00	na	na
65	Failure to transcribe special prescribed instructions to follow appropriate aseptic techniques during the preparation of a dose of a medication	8.0	3.00	8.0	2.0
66	Failure to transcribe special prescribed instructions to adhere to generally followed policies of washing hands before the preparation of the medication to be administered to the patient	7.0	4.00	7.0	2.0
67	Failure to transcribe special prescribed instructions to adhere to generally followed policies of disinfecting vials before the preparation of the medication to be administered to the patient	7.0	3.00	7.0	1.0
68	Failure to transcribe special prescribed instructions to adhere to generally followed policies of shaking the suspension bottle before administering the medication to the patient	7.0	3.00	7.0	1.0
69	Failure to transcribe special prescribed instructions to ensure the administration of a sugar-free preparation of a medication to a diabetic patient	8.0	2.00	na	na

*IQR* Interquartile range, *MTE* Medication transcription error, *na* not applicable

### Scenarios that were not included as MTE situations or remained equivocal

Analysis of the scores obtained in the second Delphi round showed that consensus was achieved to exclude 3 scenarios (3.9%) from the list of MTEs. The 3 excluded scenarios are shown in Table 4.

**Table 4:** Scenarios excluded as MTEs

#	Scenario	Round 1		Round 2	
		Median	IQR	Median	IQR
1	Transcribing a medication in its branded name as opposed to its generic (International Nonproprietary Name “INN”) or vice versa without a permission from the prescriber	3.0	3.00	3.0	2.00
2	A medication was ordered in a specified dose unit, because the nurse knew that the medication was not available in the specified dose units (strength), she/he transcribed the dose into one dose unit containing double strength and added instructions to crush the dose unit in two halves and administer one half to the patient. For example, a medication was ordered in 250 mg dose units, the nurse transcribed the medication order in dose units of 500 mg each and added instructions to crush the dose units and administer one half to the patient when the dose was due.	3.0	3.00	3.0	2.00
3	A medication was ordered in a specified dose unit, because the nurse knew that the medication was not available in the specified dose units (strength), the nurse transcribed the dose into one dose unit containing half strength and added instructions to administer two dose units of the medication to the patient when the dose was due. For example, a medication was ordered in 500 mg dose units, the nurse transcribed the medication order in dose units of 250 mg each and added instructions to administer two dose units of the medication to the patient when the dose was due.	3.0	1.25	na	na

*IQR* Interquartile range, *MTE* Medication transcription error, *na* not applicable

A total of 4 scenarios (5.3%) remained equivocal that were judged to be considered as MTE situations or not depending on the individual clinical situation which might necessitate an evaluation on case by case basis. The 4 scenarios are shown in Table 5.

**Table 5:** Scenarios to be considered as MTE situations or not depending on the clinical situation

#	Scenario	Round 1		Round 2	
		Median	IQR	Median	IQR
1	Transcribing a misspelled medication’s name (minor), however, the medication can still be recognized without confusion	5.0	4.00	5.0	4.00
2	Omission of the transcriber’s signature	5.5	3.00	5.0	4.00
3	Failure to transcribe a medication order by a nurse himself or herself	7.0	4.00	6.0	3.00
4	Failure to transcribe a medication order based on professional judgement	7.0	5.00	7.0	5.00

*IQR* Interquartile range, *MTE* Medication transcription error

## Discussion

Counting MTEs and reporting error rates depend on the definition of errors used. Inclusion of a larger number of situations considered as MTEs could lead to a larger number of errors and subsequently error rate. Previous studies addressed MTEs using definitions either developed by the researchers themselves or adopted from another study. All potential scenarios representing MTE situations were not previously included in a single study. Some scenarios potentially representing MTE situations were divisive among researchers. Numbers and/or rates of errors can be affected by the definition of errors used, detection method, counting method, and scenarios considered as error situations. These methodological variabilities can severely affect congruence in counting and reporting errors. They can also hamper comparing the numbers and rates of errors reported in different settings or counted at different occasions or by different auditors. In this study, a definition and scenarios that should or should not be considered as MTE situations were approached by a formal consensus technique. Formal consensus was achieved to accept the developed definition, 69 scenarios were formally considered as MTEs, and 3 scenarios were not considered as MTEs. To the best of our knowledge, this is the first study in which MTEs are addressed using a formal consensus technique.

In the current study, we used a purposive sampling technique to recruit the panelists. This technique was commonly used in similar studies as prior knowledge of the subject being investigated is an essential inclusion criterion [8, 9, 16, 17, 26]. Although purposive sampling has been regarded as biased in conservative views, still the use of other randomized sampling technique was not possible given the nature of the study [26, 28, 29]. However, the panelists included had prior knowledge of MTEs. Because transcription of the medications prescribed is often performed by nurses, MTEs are most likely to be committed by nurses. In this study, the majority of the panelists included were nurses. Nurses who commit medication errors are often blamed, disciplined, litigated, and/or might lose their jobs [9, 30]. Therefore, we believe nurses are more concerned with MTEs than other healthcare professionals. However, the panel included physicians, pharmacists, and risk managers.

The panel size used in this study was larger than those used in previous similar studies [8, 9, 16, 17]. Presently, there is no agreement on the ideal number of panelists in the Delphi technique. Panels used in previous studies ranged in size from 10 to over 1000 [28]. The Delphi technique was preferred over other formal consensus methods like nominal and focused groups because it maintains the anonymity of the panelists, panelists from different geographical locations can be recruited and included, reduces efforts and costs of bringing the panelists together, and reduces the possibility of one or more participants to dominate the discussion as could happen in other techniques where participants get together [28, 31].

In this study, nearly 60% of the panelists stated that there was no surveillance system for MTEs in their current institutions. These findings were not surprising and were concordant with those reported in our previous study in which about 44% of the study participants stated that there was no error surveillance system for medication administration errors in their establishments [9]. Installation of such error reporting systems was shown to be effective in reducing errors rates [32]. About half of the participants stated that their current institutions encouraged error reporting and provided clear instructions on how to minimize MTEs. However, about 60% of the participants believed the instructions and/or training in place were not enough to minimize MTEs. Again, these results were concordant with those previously reported for medication administration errors [9].

Previous studies used different definitions of MTEs [5, 7, 11, 18, 19]. Unlike the definitions of medication prescribing, dispensing and administration errors, MTEs were not approached using formal consensus techniques [8, 9, 16, 17]. The definition proposed in this study appealed to the panelists and consensus was achieved to accept this definition. Our findings in this study add to the validity and suitability of using this definition in future epidemiological studies investigating MTEs. In absence of gold standards, consensual definitions might be useful in reducing bias, promoting transparency and validity of judgmental approaches while developing certain concepts [33]. Practitioner-led definitions appeal more to researchers as well as to healthcare practitioners. Therefore, we believe this consensual definition might perform better than other definitions in future studies. Probably, nurses are more likely to change their behaviors that lead to error in response to definitions and scenarios they agree with as error situations than to definitions and scenarios they do not agree with [8, 9]. Moreover, using consensual definitions can reduce methodological variations and may permit comparing error rates in different studies and settings.

In general, consensus was achieved to include scenarios related to when the nurse transcribed a different item than the one prescribed or failed to transcribe a prescribed item, when the nurse transcribed a different dose, frequency, dosage form or count of the prescribed item, and when the nurse failed to transcribe or transcribed incorrect essential information pertaining to the prescribed item. These findings are consistent with those reported in our previous studies [8, 9]. Similarly, our results were consistent with those shown in previous studies reporting on MTEs in which incorrect transcribing of a medication’s name, dose, frequency, dosage form, notes and instructions related to the medication or dose were considered as MTEs [5, 7, 11, 18, 19].

In this study, consensus was achieved to exclude transcribing a medication using its brand-name or branded-generic name instead of the international nonproprietary name (INN) or vice versa. In Palestine as well as in many other healthcare systems, physicians prescribe using the brand-name or the name of the branded-generic instead of the INN as the use of INN is very rare. Transcribing using the brand-name or branded-generic name was justified by the panelists as a common practice instead of a MTE. Again, the panelists justified deviation from the prescribed medication dose units when the nurse added more instructions to administer the correct dose by either adding smaller units summing to the corresponding dose (strength) or crushing a higher strength dosage unit to administer the corresponding dose that was prescribed.

Misspelling a medication’s name was considered a MTE when the misspelling was major and might lead difficulty recognizing the medication. However, when the misspelling was minor and the medication’s name could be still recognized without confusion, no consensus was achieved among the panelists and the scenario remained equivocal, i.e. to be considered as MTE or not depending on the individual clinical situation. Similarly, the panelists were divisive to decide whether to consider omission of the transcriber’s signature as a MTE error or not when the guidelines require so. Again, failure to transcribe the medication order by the nurse her/himself and failure to transcribe a medication order based on professional judgement divided the opinions of the panelists. Finally, these scenarios were to be considered as MTEs or not depending on the individual clinical situation.

The findings of this study can be interpreted taking into consideration the following limitations. First, we rephrased a definition based on the definitions provided by the interviewed key contacts and the definitions found in the literature. In this study, we did not collect all the definitions either provided by each interviewee or those found in the literature and subjected them to the Delphi iterative process to choose one of them. Second, the scenarios presented to the panel in this study occur in hospitalized patient settings, therefore, our definition and scenarios apply to hospital settings and not necessarily to nursing homes. Third, the majority of the panelists in this study were nurses, findings might have been different if more physicians, pharmacists and other stakeholders were included in the panel. However, we intentionally recruited nurses as medication transcription is the responsibility of nurses in many healthcare systems. Finally, our findings might not necessarily apply to other healthcare systems in which medication transcription is not the duty of nurses or under certain legislations where other healthcare professionals have different duties.

## Conclusions

In conclusion, consensus was achieved on a definition of MTEs by a panel of healthcare professionals of whom the majority was nurses. Consensus was also achieved on scenarios that represented different MTE situations. The definition described a MTE as any discrepancy between the physician medication order and the medication order transcribed onto any document related to the patient concerned as the medical record, medication chart, medication request sheet, discharge medication chart or any other similar document. This study showed that it was possible to achieve consensus on a definition and scenarios that represent MTE situations using formal consensus techniques. Such consensual definitions could be useful in future epidemiological studies investigating MTEs.

## Additional file

Additional file 1: The questionnaire used in the Delphi rounds. (DOCX 29 kb)

## Data Availability

Data related to this study are either presented in the results section or in the additional file. Raw data can be obtained from the corresponding author on reasonable request.

## Abbreviations

BSc: Bachelor of Science
INN: International nonproprietary name
IQR: Interquartile range
IRB: Institutional review board
MD: Doctor of Medicine
MTEs: Medication transcription errors
PhD: Doctor of Philosophy
Q1: First quartile
Q2: Second quartile (median)
Q3: Third quartile
