# Supplementary material for: Medication transcription errors in hospitalized patient settings: a consensual study in the Palestinian nursing practice
Source: BMC Health Serv Res. 2019 Sep 6;19:644. doi: 10.1186/s12913-019-4485-3 (PMC6729077; doi:10.1186/s12913-019-4485-3)
Supplement: Supplementary file 1 — The questionnaire used in the Delphi rounds. (DOCX 29 kb) [file 12913_2019_4485_MOESM1_ESM.docx]

**Additional file 1: The questionnaire used in the Delphi rounds**

The study tool used in the Delphi rounds is shown below.

**Part 1: sociodemographic and practice details**

In this section, please provide your sociodemographic and practice details.

1. Gender: 🞎 Male 🞎 Female
2. Age in years: ………………………
3. Academic qualifications/degrees: 🞎 Diploma 🞎 BSc 🞎 MD 🞎 PhD 🞎 Others, please specify: …………………….
4. Place of work: ……………………..
5. Employer: 🞎 Governmental hospital 🞎 Teaching hospital 🞎 Private hospital
6. Job title: 🞎 Staff nurse 🞎 Head nurse 🞎 Physician 🞎 Pharmacist 🞎 Others, please specify: ………..
7. Number of years in practice: …………………

**Part 2: Surveillance of medication transcription errors:**

In this section, please answer the following questions on the existence of medication transcription errors surveillance system in your institution.

1. Is there any MTE surveillance system in your current institution? 🞎 Yes 🞎 No 🞎 I am not sure
2. Does your current institution encourage MTE reporting? 🞎 Yes 🞎 No 🞎 I am not sure
3. Do you receive clear instructions and/or training from the management of your institution to minimize MTEs? 🞎 Yes 🞎 No 🞎 I am not sure
4. Do you think that the instructions and/or training in place are enough to reduce MTEs? 🞎 Yes 🞎 No 🞎 I am not sure

**Part 3: Definition of medication transcription errors:**

Please indicate the degree to which you disagree or agree with the proposed definition of medication transcription errors on a scale of 1-9 (1 indicates total disagreement and 9 indicates total agreement).

| **A medication transcription error is any discrepancy between the physician medication order and the medication order transcribed onto any document related to the patient concerned as the medical record, medication chart, medication request sheet, discharge medication chart or any other similar document.** | | | | | | | | |
| --- | --- | --- | --- | --- | --- | --- | --- | --- |
| Disagreement | | | Indecisive | | | Agreement | | |
| 1 | 2 | 3 | 4 | 5 | 6 | 7 | 8 | 9 |
| Comment to justify/qualify your score (optional): | | | | | | | | |

**Part 4: Scenarios representing medication transcription error situations**

Please indicate the degree to which you disagree or agree that each of the proposed scenario represent a medication transcription error on a scale of 1-9 (1 indicates total disagreement and 9 indicates total agreement).

| 1. Transcribing a completely different medication; e.g. ibuprofen instead of metronidazole | | | | | | | | |
| --- | --- | --- | --- | --- | --- | --- | --- | --- |
| Disagreement | | | Indecisive | | | Agreement | | |
| 1 | 2 | 3 | 4 | 5 | 6 | 7 | 8 | 9 |
| Comment to justify/qualify your score (optional): | | | | | | | | |
| 1. Transcribing a look-like medication; e.g. motilium instead of movalis | | | | | | | | |
| Disagreement | | | Indecisive | | | Agreement | | |
| 1 | 2 | 3 | 4 | 5 | 6 | 7 | 8 | 9 |
| Comment to justify/qualify your score (optional): | | | | | | | | |
| 1. Transcribing a medication in its branded name as opposed to its generic (International Nonproprietary Name "INN") or *vice versa* without a permission from the prescriber | | | | | | | | |
| Disagreement | | | Indecisive | | | Agreement | | |
| 1 | 2 | 3 | 4 | 5 | 6 | 7 | 8 | 9 |
| Comment to justify/qualify your score (optional): | | | | | | | | |
| 1. Transcribing a sound-like medication; e.g. sintrom instead of centrum | | | | | | | | |
| Disagreement | | | Indecisive | | | Agreement | | |
| 1 | 2 | 3 | 4 | 5 | 6 | 7 | 8 | 9 |
| Comment to justify/qualify your score (optional): | | | | | | | | |
| 1. Failure to transcribe the dose of a medication that was prescribed to the patient | | | | | | | | |
| Disagreement | | | Indecisive | | | Agreement | | |
| 1 | 2 | 3 | 4 | 5 | 6 | 7 | 8 | 9 |
| Comment to justify/qualify your score (optional): | | | | | | | | |
| 1. Transcribing micrograms into milligrams or *vice versa* | | | | | | | | |
| Disagreement | | | Indecisive | | | Agreement | | |
| 1 | 2 | 3 | 4 | 5 | 6 | 7 | 8 | 9 |
| Comment to justify/qualify your score (optional): | | | | | | | | |
| 1. Transcribing 0.x mL into x mL or *vice versa* | | | | | | | | |
| Disagreement | | | Indecisive | | | Agreement | | |
| 1 | 2 | 3 | 4 | 5 | 6 | 7 | 8 | 9 |
| Comment to justify/qualify your score (optional): | | | | | | | | |
| 1. Transcribing x.0 mg or x.0 mL into x0 mg or x0 mL or *vice versa* | | | | | | | | |
| Disagreement | | | Indecisive | | | Agreement | | |
| 1 | 2 | 3 | 4 | 5 | 6 | 7 | 8 | 9 |
| Comment to justify/qualify your score (optional): | | | | | | | | |
| 1. Transcribing a dose of medication that is lower than the dose prescribed | | | | | | | | |
| Disagreement | | | Indecisive | | | Agreement | | |
| 1 | 2 | 3 | 4 | 5 | 6 | 7 | 8 | 9 |
| Comment to justify/qualify your score (optional): | | | | | | | | |
| 1. Transcribing a dose of medication that is higher than the dose prescribed | | | | | | | | |
| Disagreement | | | Indecisive | | | Agreement | | |
| 1 | 2 | 3 | 4 | 5 | 6 | 7 | 8 | 9 |
| Comment to justify/qualify your score (optional): | | | | | | | | |
| 1. Transcribing a smaller number of dose units than prescribed | | | | | | | | |
| Disagreement | | | Indecisive | | | Agreement | | |
| 1 | 2 | 3 | 4 | 5 | 6 | 7 | 8 | 9 |
| Comment to justify/qualify your score (optional): | | | | | | | | |
| 1. Transcribing a larger number of dose units than prescribed | | | | | | | | |
| Disagreement | | | Indecisive | | | Agreement | | |
| 1 | 2 | 3 | 4 | 5 | 6 | 7 | 8 | 9 |
| Comment to justify/qualify your score (optional): | | | | | | | | |
| 1. Transcribing a dosage form that is different from the one prescribed; e.g. capsules instead of suppositories or *vice versa* | | | | | | | | |
| Disagreement | | | Indecisive | | | Agreement | | |
| 1 | 2 | 3 | 4 | 5 | 6 | 7 | 8 | 9 |
| Comment to justify/qualify your score (optional): | | | | | | | | |
| 1. A medication was ordered in a specified dose unit, because the nurse knew that the medication was not available in the specified dose units (strength), she/he transcribed the dose into one dose unit containing double strength and added instructions to crush the dose unit in two halves and administer one half to the patient. For example, a medication was ordered in 250 mg dose units, the nurse transcribed the medication order in dose units of 500 mg each and added instructions to crush the dose units and administer one half to the patient when the dose was due. | | | | | | | | |
| Disagreement | | | Disagreement | | | Disagreement | | |
| 1 | 1 | 1 | 1 | 1 | 1 | 1 | 1 | 1 |
| Comment to justify/qualify your score (optional): | | | | | | | | |
| 1. A medication was ordered in a specified dose unit, because the nurse knew that the medication was not available in the specified dose units (strength), the nurse transcribed the dose into one dose unit containing half strength and added instructions to administer two dose units of the medication to the patient when the dose was due. For example, a medication was ordered in 500 mg dose units, the nurse transcribed the medication order in dose units of 250 mg each and added instructions to administer two dose units of the medication to the patient when the dose was due. | | | | | | | | |
| Disagreement | | | Disagreement | | | Disagreement | | |
| 1 | 1 | 1 | 1 | 1 | 1 | 1 | 1 | 1 |
| Comment to justify/qualify your score (optional): | | | | | | | | |
| 1. Transcribing a release form that is different from the one prescribed; e.g. a modified release formulation instead of conventional or *vice versa* | | | | | | | | |
| Disagreement | | | Disagreement | | | Disagreement | | |
| 1 | 1 | 1 | 1 | 1 | 1 | 1 | 1 | 1 |
| Comment to justify/qualify your score (optional): | | | | | | | | |
| 1. Failure to transcribe the frequency at which the drug was prescribed to be given | | | | | | | | |
| Disagreement | | | Indecisive | | | Agreement | | |
| 1 | 2 | 3 | 4 | 5 | 6 | 7 | 8 | 9 |
| Comment to justify/qualify your score (optional): | | | | | | | | |
| 1. Transcribing a frequency of medication administration that is lower than the one prescribed; e.g. two times a day instead of three times a day | | | | | | | | |
| Disagreement | | | Indecisive | | | Agreement | | |
| 1 | 2 | 3 | 4 | 5 | 6 | 7 | 8 | 9 |
| Comment to justify/qualify your score (optional): | | | | | | | | |
| 1. Transcribing a frequency of medication administration that is higher than the one prescribed; e.g. three times a day instead of two times a day | | | | | | | | |
| Disagreement | | | Indecisive | | | Agreement | | |
| 1 | 2 | 3 | 4 | 5 | 6 | 7 | 8 | 9 |
| Comment to justify/qualify your score (optional): | | | | | | | | |
| 1. Failure to transcribe prescribed instructions to administer a medication in relation to meals | | | | | | | | |
| Disagreement | | | Indecisive | | | Agreement | | |
| 1 | 2 | 3 | 4 | 5 | 6 | 7 | 8 | 9 |
| Comment to justify/qualify your score (optional): | | | | | | | | |
| 1. Transcribing prescribed instructions to administer a medication before meal for a medication that was prescribed to be administered after meal or *vice versa* | | | | | | | | |
| Disagreement | | | Indecisive | | | Agreement | | |
| 1 | 2 | 3 | 4 | 5 | 6 | 7 | 8 | 9 |
| Comment to justify/qualify your score (optional): | | | | | | | | |
| 1. Failure to transcribe prescribed notes on how to administer a medication | | | | | | | | |
| Disagreement | | | Indecisive | | | Agreement | | |
| 1 | 2 | 3 | 4 | 5 | 6 | 7 | 8 | 9 |
| Comment to justify/qualify your score (optional): | | | | | | | | |
| 1. Failure to transcribe the prescribed the maximum daily dose of a medication that was prescribed to be administered when required | | | | | | | | |
| Disagreement | | | Indecisive | | | Agreement | | |
| 1 | 2 | 3 | 4 | 5 | 6 | 7 | 8 | 9 |
| Comment to justify/qualify your score (optional): | | | | | | | | |
| 1. Failure to transcribe prescribed notes pertaining to special warnings or precautions concerning the administration of a medication | | | | | | | | |
| Disagreement | | | Indecisive | | | Agreement | | |
| 1 | 2 | 3 | 4 | 5 | 6 | 7 | 8 | 9 |
| Comment to justify/qualify your score (optional): | | | | | | | | |
| 1. Failure to transcribe prescribed special instructions pertaining to avoiding certain foods along with the prescribed medication | | | | | | | | |
| Disagreement | | | Indecisive | | | Agreement | | |
| 1 | 2 | 3 | 4 | 5 | 6 | 7 | 8 | 9 |
| Comment to justify/qualify your score (optional): | | | | | | | | |
| 1. Failure to transcribe prescribed special instructions pertaining to avoiding certain drugs along with the prescribed medication | | | | | | | | |
| Disagreement | | | Indecisive | | | Agreement | | |
| 1 | 2 | 3 | 4 | 5 | 6 | 7 | 8 | 9 |
| Comment to justify/qualify your score (optional): | | | | | | | | |
| 1. Failure to re-transcribe a medication order in full when a change has been made to it | | | | | | | | |
| Disagreement | | | Indecisive | | | Agreement | | |
| 1 | 2 | 3 | 4 | 5 | 6 | 7 | 8 | 9 |
| Comment to justify/qualify your score (optional): | | | | | | | | |
| 1. Failure to transcribe prescribed instructions pertaining to when to start administering a medication | | | | | | | | |
| Disagreement | | | Indecisive | | | Agreement | | |
| 1 | 2 | 3 | 4 | 5 | 6 | 7 | 8 | 9 |
| Comment to justify/qualify your score (optional): | | | | | | | | |
| 1. Failure to transcribe prescribed instructions pertaining to when to stop administering a medication | | | | | | | | |
| Disagreement | | | Indecisive | | | Agreement | | |
| 1 | 2 | 3 | 4 | 5 | 6 | 7 | 8 | 9 |
| Comment to justify/qualify your score (optional): | | | | | | | | |
| 1. Failure to transcribe a prescribed medication | | | | | | | | |
| Disagreement | | | Indecisive | | | Agreement | | |
| 1 | 2 | 3 | 4 | 5 | 6 | 7 | 8 | 9 |
| Comment to justify/qualify your score (optional): | | | | | | | | |
| 1. Transcribing a medication that was not prescribed | | | | | | | | |
| Disagreement | | | Indecisive | | | Agreement | | |
| 1 | 2 | 3 | 4 | 5 | 6 | 7 | 8 | 9 |
| Comment to justify/qualify your score (optional): | | | | | | | | |
| 1. Transcribing a medication based on an expired medication order | | | | | | | | |
| Disagreement | | | Indecisive | | | Agreement | | |
| 1 | 2 | 3 | 4 | 5 | 6 | 7 | 8 | 9 |
| Comment to justify/qualify your score (optional): | | | | | | | | |
| 1. Transcribing a medication that was put on hold | | | | | | | | |
| Disagreement | | | Indecisive | | | Agreement | | |
| 1 | 2 | 3 | 4 | 5 | 6 | 7 | 8 | 9 |
| Comment to justify/qualify your score (optional): | | | | | | | | |
| 1. Transcribing a medication that was cancelled | | | | | | | | |
| Disagreement | | | Indecisive | | | Agreement | | |
| 1 | 2 | 3 | 4 | 5 | 6 | 7 | 8 | 9 |
| Comment to justify/qualify your score (optional): | | | | | | | | |
| 1. Transcribing a wrong date | | | | | | | | |
| Disagreement | | | Indecisive | | | Agreement | | |
| 1 | 2 | 3 | 4 | 5 | 6 | 7 | 8 | 9 |
| Comment to justify/qualify your score (optional): | | | | | | | | |
| 1. Transcribing a wrong ward | | | | | | | | |
| Disagreement | | | Indecisive | | | Agreement | | |
| 1 | 2 | 3 | 4 | 5 | 6 | 7 | 8 | 9 |
| Comment to justify/qualify your score (optional): | | | | | | | | |
| 1. Transcribing a wrong patient name | | | | | | | | |
| Disagreement | | | Indecisive | | | Agreement | | |
| 1 | 2 | 3 | 4 | 5 | 6 | 7 | 8 | 9 |
| Comment to justify/qualify your score (optional): | | | | | | | | |
| 1. Failure to transcribe prescribed special instructions pertaining to storage conditions of a medication | | | | | | | | |
| Disagreement | | | Indecisive | | | Agreement | | |
| 1 | 2 | 3 | 4 | 5 | 6 | 7 | 8 | 9 |
| Comment to justify/qualify your score (optional): | | | | | | | | |
| 1. Failure to transcribe prescribed instructions to use special measuring tools to measure the dose of medication to be administered | | | | | | | | |
| Disagreement | | | Indecisive | | | Agreement | | |
| 1 | 2 | 3 | 4 | 5 | 6 | 7 | 8 | 9 |
| Comment to justify/qualify your score (optional): | | | | | | | | |
| 1. Failure to transcribe prescribed special instructions on how to reconstitute (prepare) a medication | | | | | | | | |
| Disagreement | | | Indecisive | | | Agreement | | |
| 1 | 2 | 3 | 4 | 5 | 6 | 7 | 8 | 9 |
| Comment to justify/qualify your score (optional): | | | | | | | | |
| 1. Failure to transcribe prescribed special instructions to disinfect a vial before administration of a medication | | | | | | | | |
| Disagreement | | | Indecisive | | | Agreement | | |
| 1 | 2 | 3 | 4 | 5 | 6 | 7 | 8 | 9 |
| Comment to justify/qualify your score (optional): | | | | | | | | |
| 1. Transcribing a route of drug administration that is different to that prescribed | | | | | | | | |
| Disagreement | | | Indecisive | | | Agreement | | |
| 1 | 2 | 3 | 4 | 5 | 6 | 7 | 8 | 9 |
| Comment to justify/qualify your score (optional): | | | | | | | | |
| 1. Transcribing a site of drug administration that was different to that prescribed | | | | | | | | |
| Disagreement | | | Indecisive | | | Agreement | | |
| 1 | 2 | 3 | 4 | 5 | 6 | 7 | 8 | 9 |
| Comment to justify/qualify your score (optional): | | | | | | | | |
| 1. Transcribing a medication order twice | | | | | | | | |
| Disagreement | | | Indecisive | | | Agreement | | |
| 1 | 2 | 3 | 4 | 5 | 6 | 7 | 8 | 9 |
| Comment to justify/qualify your score (optional): | | | | | | | | |
| 1. Transcribing a misspelled medication's name (minor), however, the medication can still be recognized without confusion | | | | | | | | |
| Disagreement | | | Indecisive | | | Agreement | | |
| 1 | 2 | 3 | 4 | 5 | 6 | 7 | 8 | 9 |
| Comment to justify/qualify your score (optional): | | | | | | | | |
| 1. Transcribing a misspelled medication's name (major), the medication can be confused with another | | | | | | | | |
| Disagreement | | | Indecisive | | | Agreement | | |
| 1 | 2 | 3 | 4 | 5 | 6 | 7 | 8 | 9 |
| Comment to justify/qualify your score (optional): | | | | | | | | |
| 1. Transcribing a medication order illegibly | | | | | | | | |
| Disagreement | | | Indecisive | | | Agreement | | |
| 1 | 2 | 3 | 4 | 5 | 6 | 7 | 8 | 9 |
| Comment to justify/qualify your score (optional): | | | | | | | | |
| 1. Transcribing a medication order using abbreviations or non-standard nomenclature | | | | | | | | |
| Disagreement | | | Indecisive | | | Agreement | | |
| 1 | 2 | 3 | 4 | 5 | 6 | 7 | 8 | 9 |
| Comment to justify/qualify your score (optional): | | | | | | | | |
| 1. Failure to transcribe a prescribed duration for an intravenous infusion | | | | | | | | |
| Disagreement | | | Indecisive | | | Agreement | | |
| 1 | 2 | 3 | 4 | 5 | 6 | 7 | 8 | 9 |
| Comment to justify/qualify your score (optional): | | | | | | | | |
| 1. In case the prescriber made a medication order for a patient on mg/kg basis and prescribed instructions to calculate the dose, failure to transcribe the finally calculated dose of medication on the patient's documents | | | | | | | | |
| Disagreement | | | Indecisive | | | Agreement | | |
| 1 | 2 | 3 | 4 | 5 | 6 | 7 | 8 | 9 |
| Comment to justify/qualify your score (optional): | | | | | | | | |
| 1. In case the prescriber made a medication order for a patient with renal impairment and prescribed instructions to calculate the dose, failure to transcribe the finally calculated dose of medication on the patient's documents | | | | | | | | |
| Disagreement | | | Indecisive | | | Agreement | | |
| 1 | 2 | 3 | 4 | 5 | 6 | 7 | 8 | 9 |
| Comment to justify/qualify your score (optional): | | | | | | | | |
| 1. Continuing to transcribe a medication for longer duration than instructed by the prescriber | | | | | | | | |
| Disagreement | | | Indecisive | | | Agreement | | |
| 1 | 2 | 3 | 4 | 5 | 6 | 7 | 8 | 9 |
| Comment to justify/qualify your score (optional): | | | | | | | | |
| 1. Failure to transcribe prescribed notes on the presence of other clinical conditions that might affect decisions to administer future doses of the medication prescribed to the patient | | | | | | | | |
| Disagreement | | | Indecisive | | | Agreement | | |
| 1 | 2 | 3 | 4 | 5 | 6 | 7 | 8 | 9 |
| Comment to justify/qualify your score (optional): | | | | | | | | |
| 1. Failure to transcribe prescribed information on the presence of allergies that might affect decisions to administer future doses of the medication prescribed to the patient | | | | | | | | |
| Disagreement | | | Indecisive | | | Agreement | | |
| 1 | 2 | 3 | 4 | 5 | 6 | 7 | 8 | 9 |
| Comment to justify/qualify your score (optional): | | | | | | | | |
| 1. Failure to transcribe prescribed information on contraindications that might affect decisions to administer future doses of the medication prescribed to the patient | | | | | | | | |
| Disagreement | | | Indecisive | | | Agreement | | |
| 1 | 2 | 3 | 4 | 5 | 6 | 7 | 8 | 9 |
| Comment to justify/qualify your score (optional): | | | | | | | | |
| 1. Failure to transcribe prescribed information on possibility of drug-drug interaction that might affect decisions to administer doses of the medication prescribed to the patient | | | | | | | | |
| Disagreement | | | Indecisive | | | Agreement | | |
| 1 | 2 | 3 | 4 | 5 | 6 | 7 | 8 | 9 |
| Comment to justify/qualify your score (optional): | | | | | | | | |
| 1. Failure to transcribe prescribed instructions to avoid administering certain medications along with the prescribed medication to the patient | | | | | | | | |
| Disagreement | | | Indecisive | | | Agreement | | |
| 1 | 2 | 3 | 4 | 5 | 6 | 7 | 8 | 9 |
| Comment to justify/qualify your score (optional): | | | | | | | | |
| 1. Failure to transcribe prescribed notes on possibility of drug-food interactions that might affect decisions to administer doses of the medication of the medication prescribed to the patient | | | | | | | | |
| Disagreement | | | Indecisive | | | Agreement | | |
| 1 | 2 | 3 | 4 | 5 | 6 | 7 | 8 | 9 |
| Comment to justify/qualify your score (optional): | | | | | | | | |
| 1. Failure to transcribe prescribed instructions to avoid giving the patient certain foods along with the prescribed medication | | | | | | | | |
| Disagreement | | | Indecisive | | | Agreement | | |
| 1 | 2 | 3 | 4 | 5 | 6 | 7 | 8 | 9 |
| Comment to justify/qualify your score (optional): | | | | | | | | |
| 1. Failure to transcribe special prescribed instructions on when to administer a medication; e.g. instructions to administer a dose of medication when the patient's blood pressure drops below a certain limit | | | | | | | | |
| Disagreement | | | Indecisive | | | Agreement | | |
| 1 | 2 | 3 | 4 | 5 | 6 | 7 | 8 | 9 |
| Comment to justify/qualify your score (optional): | | | | | | | | |
| 1. Failure to transcribe special prescribed instructions on when not to administer a medication; e.g. instructions not to administer a dose of medication when the patient's heart rate drops below a certain limit | | | | | | | | |
| Disagreement | | | Indecisive | | | Agreement | | |
| 1 | 2 | 3 | 4 | 5 | 6 | 7 | 8 | 9 |
| Comment to justify/qualify your score (optional): | | | | | | | | |
| 1. Failure to transcribe special prescribed instructions on monitoring the patient for certain signs before or after administering a medication; e.g. certain adverse effects | | | | | | | | |
| Disagreement | | | Indecisive | | | Agreement | | |
| 1 | 2 | 3 | 4 | 5 | 6 | 7 | 8 | 9 |
| Comment to justify/qualify your score (optional): | | | | | | | | |
| 1. Failure to transcribe special prescribed instructions on using appropriate diluents in the preparation of a dose of a medication | | | | | | | | |
| Disagreement | | | Indecisive | | | Agreement | | |
| 1 | 2 | 3 | 4 | 5 | 6 | 7 | 8 | 9 |
| Comment to justify/qualify your score (optional): | | | | | | | | |
| 1. Failure to transcribe special prescribed instructions on using appropriate solvents in the preparation of a dose of a medication | | | | | | | | |
| Disagreement | | | Indecisive | | | Agreement | | |
| 1 | 2 | 3 | 4 | 5 | 6 | 7 | 8 | 9 |
| Comment to justify/qualify your score (optional): | | | | | | | | |
| 1. Failure to transcribe special prescribed instructions to avoid mixing physically incompatible ingredients for the preparation of a dose of a medication | | | | | | | | |
| Disagreement | | | Indecisive | | | Agreement | | |
| 1 | 2 | 3 | 4 | 5 | 6 | 7 | 8 | 9 |
| Comment to justify/qualify your score (optional): | | | | | | | | |
| 1. Failure to transcribe special prescribed instructions to avoid mixing chemically incompatible ingredients for the preparation of a dose of a medication | | | | | | | | |
| Disagreement | | | Indecisive | | | Agreement | | |
| 1 | 2 | 3 | 4 | 5 | 6 | 7 | 8 | 9 |
| Comment to justify/qualify your score (optional): | | | | | | | | |
| 1. Failure to transcribe special prescribed instructions to avoid subjecting light-sensitive ingredients to light during the preparation of a dose of a medication | | | | | | | | |
| Disagreement | | | Indecisive | | | Agreement | | |
| 1 | 2 | 3 | 4 | 5 | 6 | 7 | 8 | 9 |
| Comment to justify/qualify your score (optional): | | | | | | | | |
| 1. Failure to transcribe special prescribed instructions to avoid subjecting heat-sensitive ingredients to heat during the preparation of a dose of a medication | | | | | | | | |
| Disagreement | | | Indecisive | | | Agreement | | |
| 1 | 2 | 3 | 4 | 5 | 6 | 7 | 8 | 9 |
| Comment to justify/qualify your score (optional): | | | | | | | | |
| 1. Failure to transcribe special prescribed instructions to follow appropriate aseptic techniques during the preparation of a dose of a medication | | | | | | | | |
| Disagreement | | | Indecisive | | | Agreement | | |
| 1 | 2 | 3 | 4 | 5 | 6 | 7 | 8 | 9 |
| Comment to justify/qualify your score (optional): | | | | | | | | |
| 1. Failure to transcribe special prescribed instructions to adhere to generally followed policies of washing hands before the preparation of the medication to be administered to the patient | | | | | | | | |
| Disagreement | | | Indecisive | | | Agreement | | |
| 1 | 2 | 3 | 4 | 5 | 6 | 7 | 8 | 9 |
| Comment to justify/qualify your score (optional): | | | | | | | | |
| 1. Failure to transcribe special prescribed instructions to adhere to generally followed policies of disinfecting vials before the preparation of the medication to be administered to the patient | | | | | | | | |
| Disagreement | | | Indecisive | | | Agreement | | |
| 1 | 2 | 3 | 4 | 5 | 6 | 7 | 8 | 9 |
| Comment to justify/qualify your score (optional): | | | | | | | | |
| 1. Failure to transcribe a medication order by a nurse himself or herself | | | | | | | | |
| Disagreement | | | Indecisive | | | Agreement | | |
| 1 | 2 | 3 | 4 | 5 | 6 | 7 | 8 | 9 |
| Comment to justify/qualify your score (optional): | | | | | | | | |
| 1. Failure to transcribe special prescribed instructions to adhere to generally followed policies of shaking the suspension bottle before administering the medication to the patient | | | | | | | | |
| Disagreement | | | Indecisive | | | Agreement | | |
| 1 | 2 | 3 | 4 | 5 | 6 | 7 | 8 | 9 |
| Comment to justify/qualify your score (optional): | | | | | | | | |
| 1. Failure to transcribe a medication order based on professional judgement | | | | | | | | |
| Disagreement | | | Indecisive | | | Agreement | | |
| 1 | 2 | 3 | 4 | 5 | 6 | 7 | 8 | 9 |
| Comment to justify/qualify your score (optional): | | | | | | | | |
| 1. Failure to transcribe special prescribed instructions to ensure the administration of a sugar-free preparation of a medication to a diabetic patient | | | | | | | | |
| Disagreement | | | Indecisive | | | Agreement | | |
| 1 | 2 | 3 | 4 | 5 | 6 | 7 | 8 | 9 |
| Comment to justify/qualify your score (optional): | | | | | | | | |
| 1. Omission of the transcriber's signature | | | | | | | | |
| Disagreement | | | Indecisive | | | Agreement | | |
| 1 | 2 | 3 | 4 | 5 | 6 | 7 | 8 | 9 |
| Comment to justify/qualify your score (optional): | | | | | | | | |

**Thank you very much**
